# Optimization of culture conditions for gamma-aminobutyric acid production by newly identified *Pediococcus pentosaceus* MN12 isolated from ‘mam nem’, a fermented fish sauce

**DOI:** 10.1080/21655979.2020.1857626

**Published:** 2020-12-22

**Authors:** Do Thi Bich Thuy, An Tien Nguyen, Kuan Shiong Khoo, Kit Wayne Chew, Margo Cnockaert, Peter Vandamme, Yeek-Chia Ho, Nguyen Duc Huy, Heriberto Hernández Cocoletzi, Pau Loke Show

**Affiliations:** a Hue University of Agriculture and Forestry, Hue University, Hue, Vietnam; b Faculty of Agriculture and Forestry, Dalat University, Dalat, Vietnam; c Department of Chemical and Environmental Engineering, Faculty of Science and Engineering, University of Nottingham Malaysia, Selangor Darul Ehsan, Malaysia; d School of Energy and Chemical Engineering, Xiamen University Malaysia, Jalan Sunsuria, Bandar Sunsuria, Sepang, Selangor, Malaysia; e Laboratory of Microbiology, Department of Biochemistry and Microbiology, Ghent University, Ghent, Belgium; f Civil and Environmental Engineering Department, Universiti Teknologi PETRONAS, Seri Iskandar, Perak Darul Ridzuan, Malaysia; g Centre for Urban Resource Sustainability, Institute of Self-Sustainable Building, Universiti Teknologi PETRONAS, Perak Darul Ridzuan, Malaysia; h Institute of Biotechnology, Hue University, Hue, Vietnam; i Facultad de Ingeniería Química, Benemérita Universidad Autónoma de Puebla, Pue, México

**Keywords:** GABA, optimization, *Pedioccocus pentosaceus*, fermented fish sauce

## Abstract

This study was aimed to identify and optimize the culture conditions for gamma-aminobutyric acid (GABA) production by a lactic acid bacterium strain isolated from *mam nem*, a fermented fish sauce. Among the six isolates obtained from *mam nem*, the MN12 had the most potent GABA-producing capability. The strain was then identified to be *Pedioccocus pentosaceus* by employing MALDI-TOF-MS and phenylalanyl-tRNA synthase sequencing methods. The initial cell density of 5.10^6^ CFU/mL, monosodium glutamate concentration of 60 mM, initial pH of 7, temperature of 45°C and cultivation time of 72 h were found to be the optimal culture conditions for highest production of GABA, reaching 27.9 ± 0.42 mM, by this strain. The cultivation conditions for GABA production by *P. pentosaceus* MN12 have been successfully optimized, providing a foundation for the development of fermented foods enriched with GABA.

## Graphical Abstract

**Figure:**
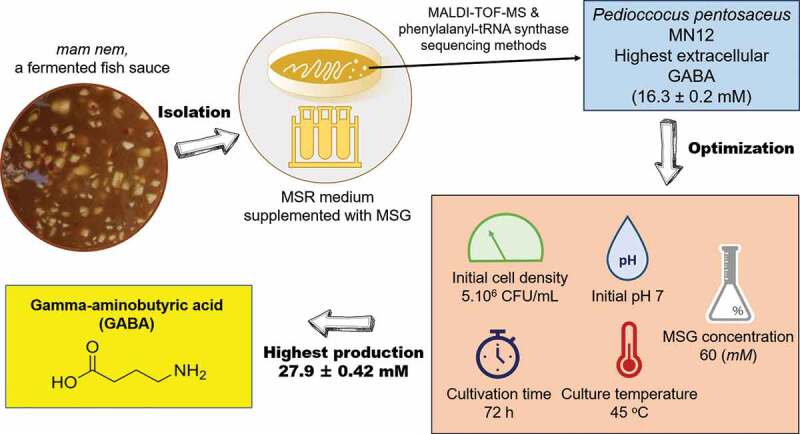

## 1. Introduction

Gamma-aminobutyric acids (GABA) are a non-protein amino acid found in bacteria, plants and animals. In mammals, GABA inhibits the neurotransmitter involving in the nervous system [1], as well as in peripheral tissues [2]. Several neurological disorders, Alzheimer’s disease and seizure for instance, are associated with the depletion of GABA in the cells [3,4]. Gamma-aminobutyric acid is also involved in multiple physiological functions, such as tumor suppression, cortisol visual improvement, cholesterol regulation, cell homeostasis maintenance and immunity enhancement [5–9]. Due to that wide range of health benefits, GABA containing foods have attracted a lot of research recently. However, common sources of food, such as fruits and vegetables, provide a relatively low level of GABA [10,11]. Therefore, fortification of foods with a considerable level of GABA from natural sources is generally desirable.

Gamma-aminobutyric acid is formed from the decarboxylation of L-glutamate by glutamate decarboxylase (GAD) [12]. Numerous studies demonstrated that GABA can be synthesized by many microorganisms including yeast, fungi and bacteria [13], with production capacity depending upon species and strains [11]. Production of GABA by microorganisms has caught much attention due to several inherent advantages, such as the fast-growing nature of microorganisms and the ease of control over the production. In the food industry, a great number of products are prepared by fermentation with the involvement of various lactic acid bacteria (LAB), many of which are promising GABA producers [13]. Therefore, LAB is a promising candidate for both food preparation and GABA fortification purposes. Many studies showed that some fermented foods, such as kimchi [14], fermented dairy [15], fermented sausage [16] and fermented black raspberry juice [11], have been successfully enriched with GABA produced by LAB. Nonetheless, it is still very important to screen various LAB species and strains that possess GABA-producing ability due to the diversity in fermentation properties of different LAB.

In Vietnam, *mam nem* is a popular sauce produced from the fermentation of fishes, particularly anchovies. The nature of the fermentation for *man nem* production is quite complex with the proteolysis being primary reactions. However, the involvement of LAB in the fermentation of *man nem* is significant as they may contribute to the taste and flavor of the product. However, LAB strains involved in *mam nem* fermentation have not been identified and their GABA-producing capacity has not been reported to our knowledge. Therefore, the present study reported the screening and the identification of the LAB strains having GABA-producing ability isolated from *mam nem*. A strain of LAB with the highest GABA yield was then selected for the optimization of the culture conditions to maximize GABA production.

## 2. Materials and methods

### 2.1. Materials

The *mam nem* were collected from local markets in Hue City, Vietnam. The Ringer’s solution was purchased from Sigma-Aldrich, while the De Man, Rogosa and Sharpe (MRS) medium were ordered from Oxoid, Milan, Italy. All chemicals used were of analytical grade.

### 2.2. Isolation of lactic acid bacteria

Samples of *mam nem* were homogenized in Ringer’s solution, serially diluted, and plated onto MRS agar and anaerobically incubated for 48 h at 37°C. The colonies were randomly picked up and transferred to new MRS agar. The subculture was repeated to obtain pure isolates. The isolates were then stained with Gram solution and tested with catalase analysis. Colonies exhibited as Gram-positive and catalase-negative were considered to be LAB, which were then stored at −80°C for further analysis.

### 2.3. Inoculant preparation

The potential LAB isolates were stationarily cultivated in capped test tubes containing 10 mL of MRS broth for 24 h at 37°C. The cell biomass was then pelleted by centrifugation at 12,000 rpm for 5 min at 4°C, followed by washing twice with Ringer’s solution. The pellet was resuspended in test tube containing 2 mL of Ringer’s solution. The cell density was spectrophotometrically measured at wavelength of 600 nm prior to use as inoculants.

### 2.4. Screening of GABA-producing LAB

The selected isolates were grown in MRS broth containing 60 mM of monosodium glutamate at initial pH of 6.2 for 24 h at 37°C. The GABA accumulated was quantified using an HPLC method mentioned below.

### 2.5. Identification of LAB strains by MALDI-TOF MS

The isolates presenting high GABA-producing capability were identified by MALDI-TOF MS method. The third-generation bacterial cells were collected after growth on MRS agar for 48 h at 37°C. Samples for MALDI-TOF MS analysis were prepared as previously reported by Freiwald and Sauer [17]. Briefly, the cells were suspended in 300 µL Milli-Q water and inactivated by adding 900 µL of absolute ethanol. After mixing, the cells were collected by centrifugation at 14,000 rpm for 3 min. The protein was extracted by resuspending the pelleted cells in 50 µL of 70% formic acid and 50 µL of acetonitrile. The protein-containing supernatant was harvested by centrifugation at 14,000 rpm for 3 min and 1 µL of solution was spotted onto a 384 Opti-TOF stainless steel MALDI target plate (AB Sciex, The Netherlands). The spot was then dried at ambient temperature and overlaid with 1 µL of acetonitrile/water/trifluoroacetic acid (50/48/2 (v/v/v)) solution containing 0.5% (w/v) α-cyano-4-hydroxycinnamic acid (α-CHCA) and subsequently dried in ambient air. The MALDI-TOF MS analysis was conducted following method described by Nguyen et al. [18]. All analyses were carried out in duplicate.

### 2.6. Molecular identification of LAB strains

The isolates that could not be identified after comparing with MS profiles of reference strains in the database were then genotypically identified by sequencing the phenylalanyl-tRNA synthase *(pheS)* gene. Total genomic DNA was extracted in the alkaline lysis buffer following a method described by Niemann et al. [19]. To amplify the *pheS* gene, a primer set of pheS-21-F (5ʹ-CAYCCNGCHCGYGAYATGC-3ʹ) and pheS-23-R (5^ʹ^-GGRTGRACCATVCCNGCHCC-3^ʹ^) were used and the PCR protocol was performed as described by Naser et al. [20]. The PCR products were sequenced, and the nucleotide sequences were aligned and compared with *pheS* sequence database using BioNumerics 7 software (Applied Math).

### 2.7. Optimization of culture conditions for GABA production

The effect of culture conditions on GABA production was evaluated using the one-factor-at-a-time approach, which means one factor was varied to estimate the effect of that factor on GABA accumulation by the LAB strain while the other factors were kept constant. The optimization parameters of interest in this study included the concentration of monosodium glutamate (0–2%), initial pH (4–9), initial cell density (5.10^5^–5.10^7^ CFU/mL), culture temperature (30°C–50°C) and culture time (24–120 h). The GABA accumulated in the culture media was quantified by using an HPLC method mentioned below.

### 2.8. Measurement of GABA content

The supernatants were obtained from MRS cultures by centrifugation at 12,000 rpm at 4°C for 5 min, and then 10-fold diluted with deionized water. Dissolved proteins were removed from the supernatants by an addition of 1 mL of 3% sulfosalicylic acid and centrifuged at 6,000 rpm for 5 min. The derivatization of GABA was carried out as described by Syu et at. [21] with minor modification. Briefly, GABA in the supernatants was derivatized by dabsylation with 4 mM 4-dimethylaminoazobenzen-4-sulfonyl chloride at 70°C for 20 min. Then, 0.5 mL of ethanol was added to the reaction mixtures and the reaction was ended by incubation in an ice bath. The dabsyl-GABA solution was centrifuged at 16,000 rpm at 4°C for 5 min and passed through 0.22 µm membrane for HPLC analysis. The HPLC system for dabsyl-GABA quantification consisted of Shimadzu LC-20A solvent delivery pump (Shimadzu, Japan) coupled with a Supelco C18 column (250 m x 4,6 mm i.d., 5 μm particle size) and a Shimadzu SPD-20A UV-Vis detector set at 465 nm wavelength. The mobile phase was 25 mM ammonium acetate buffer containing 0.1% acetic acid/acetonitrile (26/74 (v/v)). The analysis was carried out with an isocratic eluent mode with a flow rate of 1 mL/min. The column was maintained at a temperature of 55°C. A calibration curve of the GABA standard solutions at concentrations of 0, 2.4, 4.8, 7.2 and 9.6 mM was plotted and used to calculate the concentration of GABA accumulated in the culture media.

### 2.9. Statistical analysis

Data were reported as mean ± standard deviation of triplicates. One-way ANOVA was performed to detect the difference between the means, followed by Tukey’s HSD and Duncan’s multiple range tests to compare the means obtained from the screening of LAB isolates and optimization of culture condition studies, respectively. Data on time course study of pH, cell growth and GABA production were analyzed by repeated measures ANOVA. The differences were considered statistically significant at *P* ≤ 0.05. All analyses were conducted using SPSS v.16.0 (SPSS Inc, Chicago, IL USA).

## 3. Results and discussion

### 3.1. Screening of GABA-producing LAB

**Figure 1.**
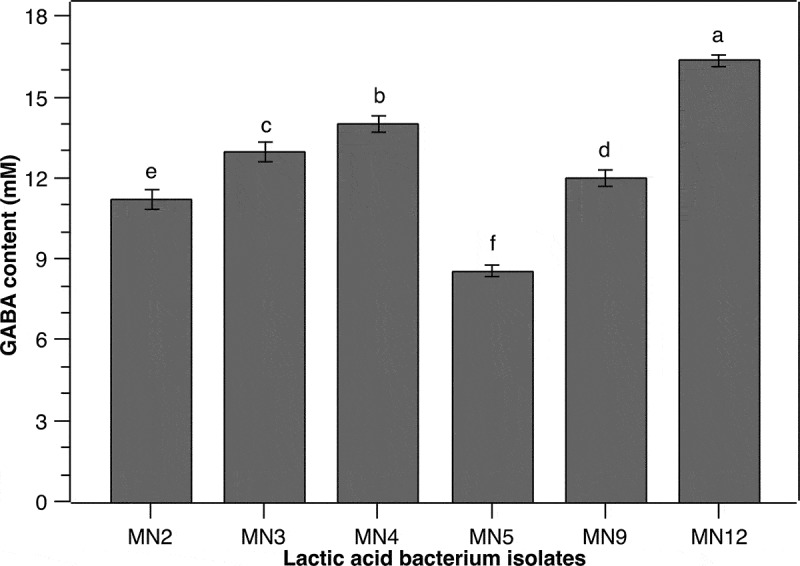
**GABA-producing capability of different LAB isolates from *mam nem.*** Cells were grown with initial cell density of 10^7^ CFU/mL in MRS broth supplemented with 60 mM of MSG for 24 h at 37°C. Concentration of GABA in culture supernatants were quantified by an HPLC method. Data are means ± SD of GABA production from triplicate experiments. Bars without a common letter differ significantly (*P* < 0.05)

The screening of GABA-producing LAB is important for further application in food industry as this group of microorganisms is a potent source of natural GABA, a bioactive agent that possesses various health benefits [22]. In this study, 30 LAB isolates have been obtained from *mam nem* samples and 6 of them presented high GABA-producing capability (Figure 1) with GABA yields ranging from around 880 to 1,680 mg/L when grown in MSR medium supplemented with 60 mM of monosodium glutamate (MSG) for 24 h at 37°C. These isolates were named as MN2, MN3, MN4, MN5, MN9, and MN12. The variation in GABA production between different isolates observed in the present study may be related to the environment-dependent GAD activity. It was reported that GABA is produced in response to environmental stresses via the alteration of GAD activity [23]. As each microorganism favors a particular environmental condition, the same culture condition used in this study may introduce different stress levels to the isolates, which impacted the GAD activity, consequently resulting in a deviation in GABA production. Nevertheless, these data emphasized the significance of LAB strains present in a traditional fermented product, particularly *mam nem*. Among the six isolates, the MN12 produced highest extracellular GABA, reaching a concentration of 16.3 ± 0.2 mM. Therefore, this isolate was selected for further studies.

### 3.2. Identification of LAB isolates

The LAB isolates classification was identified by MALDI TOF MS analysis. The resulting spectra grouped in 13 well-separated clusters with Pearson’s correlation coefficients ranging from 63.4% to 92.7%. One cluster, designated as cluster G, included 11 LAB isolates with highly similar spectra, which were obtained from *mam nem*, and other fermented foods including *nuoc mam* and *ruoc* (data not shown). These isolates remained unidentified after comparison of their MALDI TOF MS spectra with those in an *in-house* developed database. Two randomly selected isolates were identified by *pheS* sequence analysis as *Pediococcus pentosaceus* (the *pheS* gene similarity level toward a *P. pentosaceus* reference strain with NCBI accession No. AM749815 was 98% and 100%, respectively).

### 3.3. *Optimization of culture conditions for GABA production of* P. pentosaceus *MN12*

#### 3.3.1. Effect of initial cell density

**Figure 2.**
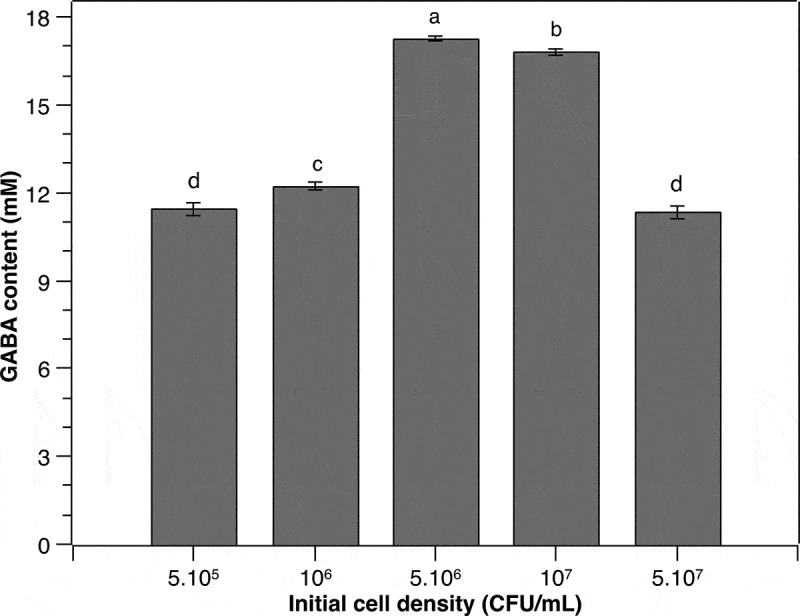
**Effect of initial cell density on the GABA production of *P. pentosaceus* MN12**. Cells were grown in MRS broth supplemented with 60 mM of MSG at 37°C for 24 h. Concentration of GABA in culture supernatants were quantified by an HPLC method. Data are means ± SD of GABA production from triplicate experiments. Bars without a common letter differ significantly (*P* < 0.05)

As individual cells function as a GABA production unit [13], the cell density obviously has a great impact on the yield of GABA. Therefore, the effect of different initial cell densities of *P. pentosaceus* MN12 on extracellular GABA accumulation in the culture was investigated in the current study. The environmental conditions including initial pH, incubation temperature, time, and MSG concentration were kept constant at 6.2, 37°C, 24 h, and 60 mM, respectively. Results showed that a maximum GABA concentration in the culture medium of 17.3 ± 0.07 mM was reached at an initial cell density of 5.10^6^ CFU/mL (Figure 2). At a lower cell density, the cells might need to adjust to a nutrient-surplus environment, which may delay the GABA peak yield. In contrast, at a higher initial cell density, stress might be imposed on the cells [24], which consequently interferes with GABA synthesis by the cells. Li et al. [25] reported that high cell density increases GABA synthesis. However, our current study clearly demonstrated that the maximum GABA yield of *P. pentosaceus* MN12 was obtained at an appropriate initial cell density. This result was in agreement with previous research of Ratanaburee et al. [26], showing that maximal amounts of GABA in a Thai fermented pork sausage were produced by *Lactobacillus namurensis* NH2 and *Pediococcus pentosaceus* HN8 at a 10^6^ CFU/g, which was relatively lower than other experimented cell densities of 10^7^ and 10^8^ CFU/g.

#### 3.3.2. Effect of glutamate concentration

**Figure 3.**
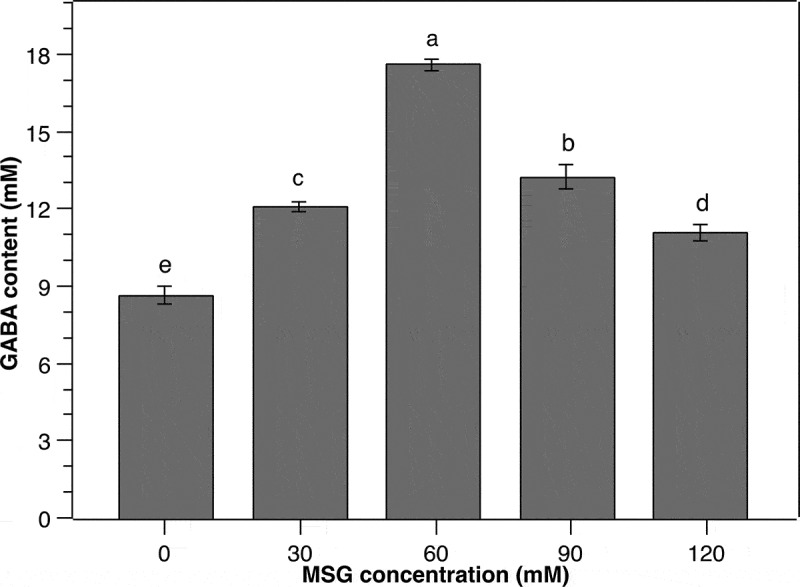
**Effect of monosodium glutamate (MSG) concentration on the GABA production of *P. pentosaceus* MN12**. Cells were grown in MRS broth with initial cell density of 5.10^6^ CFU/mL at 37°C for 24 h. Concentration of GABA in culture supernatants were quantified by an HPLC method. Data are means ± SD of GABA production from triplicate experiments. Bars without a common letter differ significantly (*P* < 0.05)

Gamma-aminobutyric acid (GABA) is produced from the bioconversion of glutamate catalyzed by GAD in many species including LAB [27]. Several microorganisms, *Corynebacterium glutamicum* for instance, have a strong amino acid-producing ability, enabling GABA production from endogenous L-glutamate [28]. However, most LAB are not capable of synthesizing enough L-glutamate for GABA production purposes. Therefore, the supplementation of MSG to the culture media is indispensable as MSG can be easily hydrolyzed to L-glutamate [13]. Herein, MSG was supplemented to the culture medium of *P. pentosaceus* MN12 at various concentrations. As a result, the content of GABA increased proportionally to the increments of MSG concentration from 0 to 60 mM (Figure 3). The GABA peak of 17.6 ± 0.24 mM was obtained in the culture medium supplemented with 60 mM of MSG. At higher MSG concentrations, however, the production of GABA by *P. pentosaceus* MN12 was reduced substantially. This reduction might be due to the elevation in osmotic pressure of the culture medium caused by increased MSG concentration, consequently disturbing the metabolism as well as GABA synthesis of the cells [22]. It was concluded from this experiment that the MSG concentration of 60 mM in culture medium was the most suitable for GABA production by this LAB strain.

#### 3.3.3. Effect of initial pH

**Figure 4.**
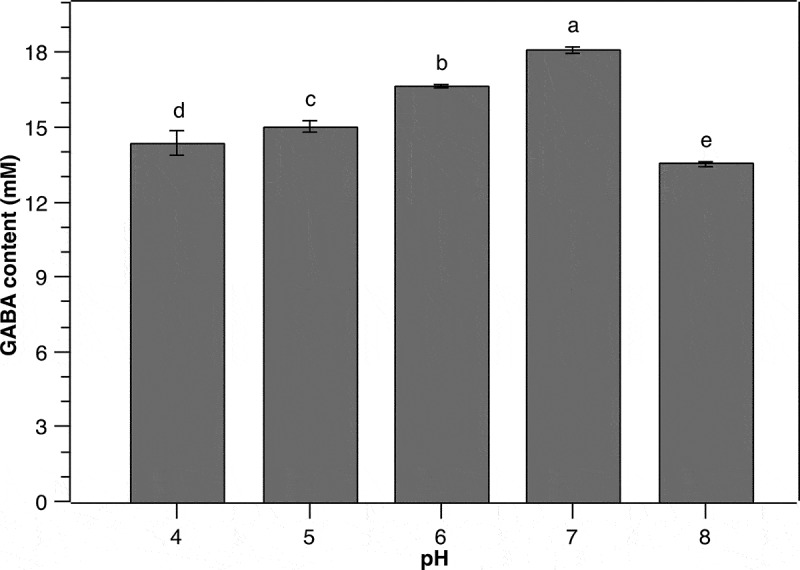
**Effect of initial pH on the GABA production of *P. pentosaceus* MN12**. Cells were grown in MRS broth with initial cell density of 5.10^6^ CFU/mL at 37°C for 24 h. Concentration of GABA in culture supernatants were quantified by an HPLC method. Data are means ± SD of GABA production from triplicate experiments. Bars without a common letter differ significantly (*P* < 0.05)

The pH of culture medium is a crucial condition affecting GABA production of different bacteria as it alters the bacterial growth and the activity of glutamate decarboxylase [11,14,25,29,30]. In addition, GABA synthesis of several LAB species has been documented to vary greatly depending upon initial pH values of the culture medium [11,25]. Therefore, in the present work, we investigated the effect of initial pH on GABA production by *P. pentosaceus* MN12. As can be seen from Figure 4, the GABA yield increased gradually from an initial pH 4 to 7 and subsequently dropped markedly at higher initial pH. At initial pH 7, the concentration of GABA in the media reached a maximum level of 18.1 ± 0.13 mM. This result indicated that an initial neutral pH was favorable for GABA accumulation by *P. pentosaceus* MN12, albeit the glutamate decarboxylase activity being highest at pH 5 [31]. It must be noted, however, the pH in the culture medium is changed during fermentation process by bacteria, consequently bringing the pH to the level more appropriate for GABA synthesis [11,29]. It was also shown in this research that higher medium pH led to a substantial reduction in GABA production level, which may be due to the inhibition of cell growth caused by an alkaline environment.

#### 3.3.4. Effect of culture temperature

**Figure 5.**
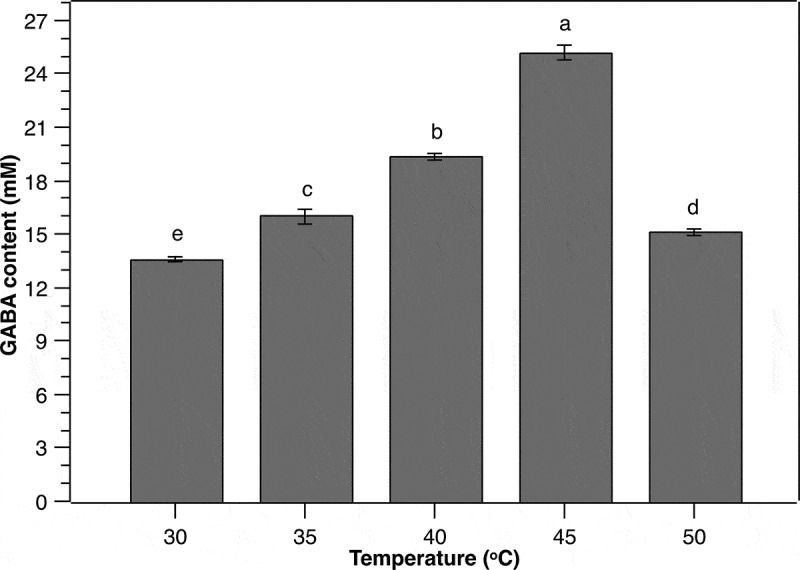
**Effect of temperature on the GABA production from *P. pentosaceus* MN12**. Cells were grown in MRS broth with initial cell density of 5.10^6^ CFU/mL at initial pH 7 for 24 h. Concentration of GABA in culture supernatants were quantified by an HPLC method. Data are means ± SD of GABA production from triplicate experiments. Bars without a common letter differ significantly (*P* < 0.05)

Temperature has been demonstrated to have strong impact on GABA production of LAB as both cell growth and GAD activity are temperature-dependent [13]. Therefore, it is essential to uncover the optimum incubation temperature for GABA accumulation by *P. pentosaceus* MN12 to maximize the yield. In the current work, the cells were cultivated in MSR broth in optimal conditions of initial cell density, MSG concentration, and initial pH as determined above, followed by GABA content monitoring after 24 h. Gamma-aminobutyric acid concentration in the medium increased proportionally with the increase of temperature from 30°C to 45°C (Figure 5). The highest extracellular GABA level of 25.1 ± 0.44 mM was obtained at 45°C, suggesting that this temperature was optimal for GABA production by *P. pentosaceus* MN12. However, the higher temperature at 50°C resulted in a marked decline in the concentration of GABA produced by the strain, indicating a negative impact of this temperature. Culture temperature affects GABA production due most likely to its influence on GAD activity. According to Yang et al. [32], GAD activity increases to a maximum in response to the increase in the temperature to a certain degree, then decreases gradually with further increase of the temperature. The pattern of GABA accumulation by the MN12 strain as a function of temperature observed in this study suggested a connection to that variation of GAD functionality. Nevertheless, further studies are needed to elucidate the relationship between the activity of *P. pentosaceus* MN12-derived GAD and temperature.

#### 3.3.5. Time course study of pH, cell growth and GABA accumulation

**Figure 6.**
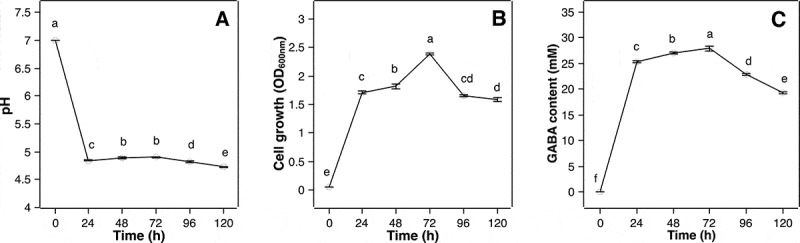
**Effect of fermentation time on pH** (a)**, cell growth** (b) **and GABA accumulation** (c) **by *P. pentosaceus* MN12**. Cells were grown in MRS broth in optimal conditions for 120 h. The measurements of pH, cell growth and GABA content were taken at 24 h intervals. Data are means ± SD from triplicate experiments. Means without a common letter differ significantly (*P* < 0.05)

A time course study was conducted to elucidate the interrelationship between the changes of pH, cell growth, and GABA accumulation by the MN12 strain with respect to time. Figure 6 shows the culture medium pH dropped from 7 to around 4.8 within the first 24 h (Figure 6(a)), concomitant with the increase in cell growth (Figure 6(b)) and extracellular GABA concentration (Figure 6(c)), suggesting that lactic acid fermentation and GABA synthesis happened concurrently. Also, this fermentation brought the pH to the level optimal for GAD, which was 4.5 to 5 [31,33]. In the next 48 h, medium pH increased slightly to around 5 with a further increase in cell growth and GABA accumulation. This slight increase in pH might be due to the consumption of H^+^ by GAD to produce GABA (net charge +1), which were exported extracellularly in exchange of glutamate (net charge 0), making the culture medium slightly more alkaline [34]. After 72 h of fermentation, the cell density reached a maximum, which corresponded to a peak of GABA production of 27.9 ± 0.42 mM. However, prolonged cultivation time beyond 72 h may lead to a depletion of nutrients in the medium, cell death and autolysis of the dead cells, which consequently resulted in a reduction in cell numbers (Figure 6(b)). In addition, due to this nutrient shortage, the extracellular GABA might be taken back to the cells and degraded to succinic semialdehyde and subsequently to succinate by the GABA aminotransferase and succinate semialdehyde dehydrogenase enzymes, respectively, for energy demand [13], leading to a decrease in GABA accumulation observed in this study (Figure 6(c)).

At optimal culture conditions, the GABA-producing ability varies greatly depending on LAB species. For example, the GABA concentration produced by *Lactobacillus acidophilus* BCRC 14,079 isolated from commercial yogurt was ~2.2 mM, whereas *Lactobacillus brevis* NL192 was able to yield ~2000 mM of GABA [13]. In this regards, *P. pentosaceus* MN12 can be considered a moderate GABA producer. However, the growth and fermentation ability of the MN12 in an environment of very high salinity might make this strain to be essential in the fermented food industry. In addition, compared with *P. pentosaceus* LMG 11,488 and *P. pentosaceus* IFK-11, which had capabilities of synthesizing GABA up to 0.58 mM and 19.98 mM [35], respectively, the MN12 strain obtained from *mam nem* as reported in this study might have a greater potential application in the food industry.

## 4. Conclusions

In summary, six GABA-producing LAB strains were successfully isolated from *mam nem* with MN12 being the most potent candidate. The MN12 isolate was then identified to be *P. pentosaceus* MN12. The optimal conditions for the highest level of GABA yielded by this strain were initial density of 5.10^6^ CFU/mL, MSG concentration 60 mM, initial pH of 7, temperature of 45°C, and cultivation time of 72 h. At the optimal conditions, a GABA content of 27.9 ± 0.42 mM was accumulated in the culture medium. To the authors’ best knowledge, this research is the first of its kind to identify *P. pentosaceus* MN12 from *mam nem*, a fermented sauce made from fish, and to optimize the GABA production of this strain by modifying culture conditions. The results from this study provide a background for further development of functional fermented foods that *P. pentosaceus* is the primary fermentation bacterium.

## Research highlights

- Six GABA-producing bacterial strains were isolated from *mam nem*, a fermented fish sauce
- The most potent strain for GABA production among the six was *Pedioccocus pentosaceus* MN12
- Culture conditions for the highest production of GABA by the strain were successfully optimized.
- The maximal yield of GABA produced by *P. pentosaceus* MN12 under optimal conditions were 27.9 ± 0.42 mM.

## References

[1] Krnjevic K. Chemical nature of synaptic transmission in vertebrates. Physiol Rev. 1974;54:418–540.

[2] DeFeudis FV. Muscimol binding and GABA receptors. Drug Dev Res [Internet]. 1981;1::93–105. Available from https://onlinelibrary.wiley.com/doi/abs/10.1002/ddr.430010202

[3] Maggio R, Sohn E, Gale K. Lack of proconvulsant action of GABA depletion in substantia nigra in several seizure models. Brain Res [Internet]. 1991;547:1–6. Available from: http://www.sciencedirect.com/science/article/pii/000689939190567F 1650283 10.1016/0006-8993(91)90567-f

[4] Seidl R, Cairns N, Singewald N, et al. Differences between GABA levels in alzheimer’s disease and down syndrome with alzheimer-like neuropathology. Naunyn Schmiedebergs Arch Pharmacol [Internet]. 2001;363:139–145. Available from 10.1007/s002100000346 11218066

[5] Abdou AM, Higashiguchi S, Horie K, et al. Relaxation and immunity enhancement effects of γ-Aminobutyric acid (GABA) administration in humans. BioFactors. 2006;26:201–208. 16971751 10.1002/biof.5520260305

[6] Imam MU, Ishaka A, Ooi D-J, et al. Germinated brown rice regulates hepatic cholesterol metabolism and cardiovascular disease risk in hypercholesterolaemic rats. J Funct Foods [Internet]. 2014 cited 2014 Oct 28;8::193–203. Available from: http://linkinghub.elsevier.com/retrieve/pii/S1756464614001091

[7] Leventhal AG, Wang Y, Pu M, et al. GABA and its agonists improved visual cortical function in senescent monkeys. Science [Internet]. 2003;300:812 LP– 815. Available from: http://science.sciencemag.org/content/300/5620/812.abstract 12730605 10.1126/science.1082874

[8] Schuller HM, HAN A-W, Majidi M. Gamma-aminobutyric acid, a potential tumor suppressor for small airway-derived lung adenocarcinoma. Carcinogenesis. 2008;29:1979–1985. 18310090 10.1093/carcin/bgn041 PMC2556972

[9] Warskulat U, Reinen A, Grether-Beck S, et al. The osmolyte strategy of normal human keratinocytes in maintaining cell homeostasis. J Invest Dermatol. 2004;123:516–521. 15304091 10.1111/j.0022-202X.2004.23313.x

[10] Fougère F, Le Rudulier D, Streeter JG. Effects of salt stress on amino acid, organic acid, and carbohydrate composition of roots, bacteroids, and cytosol of alfalfa (Medicago sativa L.). Plant Physiol [Internet]. 1991;96:1228 LP– 1236. Available from: http://www.plantphysiol.org/content/96/4/1228.abstract 16668324 10.1104/pp.96.4.1228 PMC1080920

[11] Kim JY, Lee MY, Ji GE, et al. Production of γ-aminobutyric acid in black raspberry juice during fermentation by Lactobacillus brevis GABA100. Int J Food Microbiol [Internet]. 2009;130::12–16. Available from 19167126 10.1016/j.ijfoodmicro.2008.12.028

[12] Higuchi T, Hayashi H, Abe K. Exchange of glutamate and γ-aminobutyrate in a Lactobacillus strain. J Bacteriol. 1997;179:3362–3364. 9150237 10.1128/jb.179.10.3362-3364.1997 PMC179120

[13] Cui Y, Miao K, Niyaphorn S, et al. Production of gamma-aminobutyric acid from lactic acid bacteria: A systematic review. Int J Mol Sci. 2020;21:1–21. 10.3390/ijms21030995 PMC7037312 32028587

[14] Cho SY, Park MJ, Kim KM, et al. Production of high γ-aminobutyric acid (GABA) sour kimchi using lactic acid bacteria isolated from Mukeunjee kimchi. Food Sci Biotechnol. 2011;20:403–408.

[15] Nejati F, Rizzello CG, Di Cagno R, et al. Manufacture of a functional fermented milk enriched of angiotensin-I converting enzyme (ACE)-inhibitory peptides and γ-amino butyric acid (GABA). [Internet]. LWT - Food Sci Technol. 2013;51::183–189. Available from 10.1016/j.lwt.2012.09.017

[16] Yu HH, Choi JH, Kang KM, et al. Potential of a lactic acid bacterial starter culture with gamma-aminobutyric acid (GABA) activity for production of fermented sausage. Food Sci Biotechnol. 2017;26:1333–1341. 30263667 10.1007/s10068-017-0161-8 PMC6049782

[17] Freiwald A, Sauer S. Phylogenetic classification and identification of bacteria by mass spectrometry. Nat Protoc. 2009;4:732–742. 19390529 10.1038/nprot.2009.37

[18] Nguyen DTL, Van Hoorde K, Cnockaert M, et al. A description of the lactic acid bacteria microbiota associated with the production of traditional fermented vegetables in Vietnam. [Internet]. Int J Food Microbiol. 2013;163::19–27. Available from 10.1016/j.ijfoodmicro.2013.01.024 23500611

[19] Niemann S, Pühler A, Tichy HV, et al. Evaluation of the resolving power of three different DNA fingerprinting methods to discriminate among isolates of a natural Rhizobium meliloti population. J Appl Microbiol. 1997;82:477–484. 9134721 10.1046/j.1365-2672.1997.00141.x

[20] Naser SM, Dawyndt P, Hoste B, et al. Identification of lactobacilli by pheS and rpoA gene sequence analyses. Int J Syst Evol Microbiol. 2007;57:2777–2789. 18048724 10.1099/ijs.0.64711-0

[21] Syu KY, Lin CL, Huang HC, et al. Determination of theanine, GABA, and other amino acids in green, oolong, black, and Pu-erh teas with dabsylation and high-performance liquid chromatography. J Agric Food Chem. 2008;56:7637–7643. 18652476 10.1021/jf801795m

[22] Villegas JM, Brown L, Savoy de Giori G, et al. Optimization of batch culture conditions for GABA production by Lactobacillus brevis CRL 1942, isolated from quinoa sourdough. LWT - Food Sci Technol. 2016;67:22–26.

[23] Kinnersley AM, Turano FJ. Gamma aminobutyric acid (GABA) and plant responses to stress. CRC Crit Rev Plant Sci. 2000;19:479–509.

[24] Bunch AW. High cell density growth of micro-organisms. Biotechnol Genet Eng Rev. 1994;12:535–561. 7727037 10.1080/02648725.1994.10647921

[25] Li H, Qiu T, Huang G, et al. Production of gamma-aminobutyric acid by Lactobacillus brevis NCL912 using fed-batch fermentation. Microb Cell Fact. 2010;9:1–7. 21070676 10.1186/1475-2859-9-85 PMC2996345

[26] Ratanaburee A, Kantachote D, Charernjiratrakul W, et al. Enhancement of γ-aminobutyric acid (GABA) in nham (Thai fermented pork sausage) using starter cultures of lactobacillus namurensis NH2 and Pediococcus pentosaceus HN8 [Internet]. International Journal of Food Microbiology. 2013;167(2):170–176. Elsevier B.V.Available from: 10.1016/j.ijfoodmicro.2013.09.014 24135673

[27] Diana M, Quílez J, Rafecas M. Gamma-aminobutyric acid as a bioactive compound in foods: A review. [Internet]. J Funct Foods. 2014;10::407–420. Available from 10.1016/j.jff.2014.07.004

[28] Heider SAE, Wendisch VF. Engineering microbial cell factories: metabolic engineering of Corynebacterium glutamicum with a focus on non-natural products. Biotechnol J. 2015;10:1170–1184. 26216246 10.1002/biot.201400590

[29] Di Cagno R, Mazzacane F, Rizzello CG, et al. Synthesis of γ-aminobutyric acid (GABA) by Lactobacillus plantarum DSM19463: functional grape must beverage and dermatological applications. Appl Microbiol Biotechnol. 2010;86:731–741. 20013120 10.1007/s00253-009-2370-4

[30] Cho YR, Chang JY, Chang HC. Production of gamma-aminobutyric acid (GABA) by Lactobacillus buchneri isolated from kimchi and its neuroprotective effect on neuronal cells. [Internet]. J Microbiol Biotechnol. 2007;17:104–109. Available from http://europepmc.org/abstract/MED/18051360 18051360

[31] Komatsuzaki N, Shima J, Kawamoto S, et al. Production of γ-aminobutyric acid (GABA) by Lactobacillus paracasei isolated from traditional fermented foods. Food Microbiol. 2005;22:497–504.

[32] Yang T, Rao Z, Kimani BG, et al. Two-step production of gamma-aminobutyric acid from cassava powder using Corynebacterium glutamicum and Lactobacillus plantarum. J Ind Microbiol Biotechnol. 2015;42:1157–1165. 26115763 10.1007/s10295-015-1645-2

[33] Yang SY, Lü FX, Lu ZX, et al. Production of γ-aminobutyric acid by Streptococcus salivarius subsp. thermophilus Y2 under submerged fermentation. Amino Acids. 2008;34:473–478. 17514494 10.1007/s00726-007-0544-x

[34] De Biase D, Pennacchietti E. Glutamate decarboxylase-dependent acid resistance in orally acquired bacteria: function, distribution and biomedical implications of the gadBC operon. Mol Microbiol. 2012;86:770–786. 22995042 10.1111/mmi.12020

[35] Agung Yogeswara IB, Kusumawati IGAW, Sumadewi NLU, et al. Isolation and identification of lactic acid bacteria from Indonesian fermented foods as γ-aminobutyric acid-producing bacteria. Int Food Res J. 2018;25:1753–1757.

